# News and Notes

**Published:** 1905-04

**Authors:** 


### NEWS AND NOTES.

THE number of persons who died of pellagra in Italy in 1903 was 2,648.

$100,000 has already been subscribed for the construction of the Children's Memorial Hospital, Chicago, which will cost $300,000.

THE legislature of Pennsylvania passed a bill regulating and licensing the practice of osteopaths; it also provides for the establishment of a State board of examiners.

THE Nobel prize in physical science has been awarded this year to the English physicist, Lord Rayleigh. His coworker, Sir William Ramsay, received the prize in chemistry. The five prizes now amount to $40,000 each.

THE statistics of the Naples Antirabic Institute for the three years 1901–1903 have just been published. The number of persons admitted during that period was 1,470. The percentage of deaths in the total number was two.

DR. ROBERT WOODWARD, who has been chosen head of the Carnegie Institute, has been dean of the School of Pure Science at Columbia University since 1895, and professor of mechanics and mathematical physics since 1893.

THE *Canada Medical Record*, for many years conducted by Dr. F. W. Campbell, dean of the Medical Faculty of Bishop's College University, Montreal, has ceased publication and has become amalgamated with the *Montreal Medical Journal.*

GEORGE V. N. DEARBORN, A.M. (Harvard), M.D., Ph.D. (Columbia), has been elected to the Professorship of Physiology in the Tufts College Medical and Dental Schools, Boston. More than six hundred students of both sexes are in attendance at these schools.

---

A BILL introduced by Mr. Burnett has passed the legislature of Illinois prohibiting attorneys from soliciting personal or other damage cases. This measure was endorsed by the State Bar Association, and is directed against shyster lawyers, who make a business of drumming up this character of practice against members of the medical profession.

---

A PRIVATE citizen has placed in the hands of the government of the Grand Duchy of Baden a sum of $650 toward the foundation at Heidelberg of an institute for the study of cancer. The government has given a site for the purpose in the immediate neighborhood of the University Hospital, and has promised a grant for the maintenance of the institute.

---

AMONG the members of the medical profession in foreign countries who have recently died are Dr. A. S. Herrero, professor of clinical medicine in the University of Madrid; and Dr. Alexander Bobroff, professor of surgery, director of the surgical clinic of the University of Moscow, and a former president of the Russian Surgical Society, aged fifty-four.

---

A NUMBER of actuaries and medical directors of New York insurance companies have, says a contemporary, been working on statistics running over a period of several years. From the record it is claimed that total abstainers as a class live longer by from twenty to fifty per cent than moderate drinkers, so that this class of applicants will soon have to pay less for insurance than the moderate drinkers.

---

IT is reported, says an exchange, that so great is the rush of Russian Jews arriving in London suffering from trachoma that the hospitals are turning them away. A large number of these persons are *en route* to America, while others have been rejected by the United States immigrant inspectors, and having been returned by the steamship companies are stranded in the United Kingdom instead of being sent back to Russia.

---

THE Bulletin of Chicago's Health Department states that recently the laboratory has been making a systematic examination of the air to determine the cleansing effect of snow. It was found that even a slight snowfall produced a decided reduction in the number of bacteria in the air. The greatest reduction (falling upon ten square inches of surface in a minute) noted was a decrease from 1020 on November 26 to twenty-three on December 3, produced by the slight fall of snow on the last-named date.

---

DR. JOSEPH McDONALD announces that he has severed his connection as manager and managing editor of the *International Journal of Surgery,* with which he has been associated for the past fourteen years. This move was made for the purpose of enabling him to publish an independent practical surgical journal and he has purchased all rights in the *American Journal of Surgery and Gynecology*, and with the April number of this journal, thoroughly modernized and largely increased in circulation, will be issued from New York as the *American Journal of Surgery.*

---

THE endless chain system is put into operation in checking the plague in Bombay, India. To a person coming for preventive inoculation four coupons are sold for a small fee. When he induces four persons to be inoculated, presenting these coupons, he is rewarded with a generous payment. Each one who can be induced to do so takes four coupons to work in a similar manner. This can be worked in regard to vaccination, or any other hygienic procedure in which the safety of the public is as much concerned as is that of the individual himself.

"THE Crusade Against Tuberculosis in Philadelphia" was the topic of D. L. F. Flick in an address before the Acorn Club. When asked his opinion of the danger of spitting on the sidewalks and in public places, and whether those people who expectorated in these places were not sufferers of tuberculosis, he replied to the question in the negative. He said "the poor consumptive is bearing more than his burden already and should not be made to share the responsibility of this evil." He informed the audience that a law should be passed prohiting expectorating on the sidewalks and in public places.

---

AT the anuual meeting of the Board of Trustees of the Carnegie Institute, held at Washington on December 13, it was announced, according to the *Medical Record*, that during the year $355,070 had been expended to further research in various departments of science, and that twenty-four research assistants had received $1,000 each. The projects undertaken include the establishment of a department of biology with stations at Cold Spring Harbor, L. I., and at the Dry Tortugas, Fla.; of a department of economics and sociology; a bureau of historical research; a department of international researches in terrestrial magnetism. Special grants were made for an archeological expedition to the trans-Caspian region, and for geophysical research. Other grants were for the encouragement of biological researches at the Marine Biological Laboratory, Woods Hole, Mass.; also at Dr. Dorhn's laboratory at Naples; for an anthropological investigation in the United States and archeological investigation in Syria, Palestine, Egypt, Nubia, etc.; for important astronomical investigations in several of the great observatories and elsewhere; for the publication of the *Index Medicus;* also for other important bibliographical work, such as the preparation of a handbook of learned societies of America, etc., for investigation of desert vegetation; for study of the fundamental principles of geology; for extended geological explorations in China; and for investigations in nutrition at the laboratory at Middletown, Conn.

